# Correction to “Quantification of Diffuse Parenchymal Lung Disease in Non‐Small Cell Lung Cancer Patients With Definitive Concurrent Chemoradiation Therapy for Predicting Radiation Pneumonitis”

**DOI:** 10.1111/1759-7714.70207

**Published:** 2025-12-11

Y. C. An, J. H. Kim, J. M. Noh, K. Yang, Y. J. Oh, S. G. Park, H. R. Pyo, and H. Y. Lee, “Quantification of Diffuse Parenchymal Lung Disease in Non‐Small Cell Lung Cancer Patients With Definitive Concurrent Chemoradiation Therapy for Predicting Radiation Pneumonitis,” *Thoracic Cancer* 14, no. 36 (2023): 3530–3539, https://doi.org/10.1111/1759‐7714.15156.

In the author list of the above article, one of the co‐authors' names was incorrectly given as “Kyung Mi Yang”. The correct spelling of the author's name is “Kyungmi Yang”.

The correct author information is:

Kyungmi Yang, MD, Department of Radiation Oncology, Samsung Medical Center, Sungkyunkwan University School of Medicine, Seoul, Republic of Korea

The online version of this article has been corrected accordingly.

We apologize for this error.

